# Political probity increases trust in government: Evidence from randomized survey experiments

**DOI:** 10.1371/journal.pone.0225818

**Published:** 2020-02-24

**Authors:** Aaron Martin, Raymond Orr, Kyle Peyton, Nicholas Faulkner

**Affiliations:** 1 School of Social and Political Science, The University of Melbourne, Parkville, Victoria, Australia; 2 Native American Studies Department, The University of Oklahoma, Norman, Oklahoma, United States of America; 3 Department of Political Science and Institution for Social and Policy Studies, Yale University, New Haven, Connecticut, United States of America; 4 BehaviourWorks Australia, Monash University, Clayton, Victoria, Australia; Mälardalen University, SWEDEN

## Abstract

Low levels of trust in government have potentially wide-ranging implications for governing stability, popular legitimacy, and political participation. Although there is a rich normative and empiricial literature on the important consequences of eroding trust in democratic societies, the causes of political trust are less clear. In this article we estimate the effect that changing Americans’ views about the perceived honesty and integrity of political authorities (or “political probity”) has on their trust in government using randomized survey experiments. In one experiment on a convenience sample and a direct replication on a more representative sample, we find that a single Op-Ed article about political probity increased trust in government by an amount larger than the partisan gap between Democrats and Republicans. These results complement prior observational studies on trust in government by demonstrating that political probity plays an important causal role in shaping Americans’ judgments about the trustworthiness of their government and politicians.

## Introduction

Trust in government is an essential feature of democratic society [1], and governments depend on citizens’ trust to maintain legitimacy [2–6] and implement public policy [7–10]. In recent years, declining trust in government and politicans across many advanced democracies has been associated with democratic backsliding and a rise in support for anti-system parties and politicans, such as Donald Trump and Brexit [11, 12]. Although there is a rich normative and empiricial literature on the import *consequences* of eroding trust, relatively little is known about what causes political trust, or distrust. One potential cause of political trust is “probity”, or the perceived honesty and integrity of political authorities, and a large body of survey research in the United States suggests this is one of the primary drivers of variation in political trust across individuals and time [6, 13–16]. For example, salient signals of *an absence* of political probity, such as Watergate and the House Banking Scandal, are often followed by declining political trust in national surveys [13, 15–17]. This prior research, however, draws largely on observational studies that are uniquely focused on explaining factors that *decrease* political trust. Consequently, the causal link between probity and political trust is somewhat tentative.

Here we use randomized survey experiments to investigate the consequences of providing information about the presence (or absence) of probity with putative opinion pieces (“Op-Eds”), instruments of persuasion that have strong and lasting effects on public opinion across a variety of topics [18]. In two experiments—one on Amazon Mechanical Turk (MTurk) and a direct replication on a population based online panel—we find that exposure to a single Op-Ed about the probity of political elites significantly increased political trust. Substantively, these effects were larger than the observed gap in political trust between Democrats and Republicans in control. In a third placebo experiment we find that the effects of Op-Eds about corruption in the National Football League (NFL) on political trust were negligible and not statistically distinguishable from zero, suggesting the effects in Experiments 1–2 were driven by shifting perceptions about the probity of *political* elites. Counteracting the erosion of trust in government in the United States may therefore require changing Americans’ beliefs that their politicians are dishonest and government corruption is widespread.

## Materials and methods

Experiment 1 was conducted on Amazon Mechanical Turk (MTurk) respondents (n = 643), and Experiment 2 used a sample of respondents from Qualtrics panels (n = 1,324) that was selected to approximate the general population in the United States, and Experiment 3 was conducted on MTurk (n = 584). The Qualtrics sample was collected to approximate the United States general population on age, sex, race, education and party identification. Experiments 1 and 3 used simple random assignment and Experiment 2 used stratified random assignment based on reported partisan identification (Republican, Democrat, Independent). Experiments 1–2 were otherwise identical, and Experiments 2 should therefore be viewed as a direct replication of Experiment 1 on a more representative sample. The research design was approved by the [redacted for anonymity] and all participants consented to participation in the study in writing after they were presented with a plain language statement. Participants were required to be 18 years or older. Additional details are available in the Supplementary Tables 1–10 in S1 Appendix. This research was supported by grants from the Australian Research Council (“Understanding the Causes of Political Trust, DE #160100603) and the Faculty Research Grant from the University of Oklahoma. The research was approved by the Human Research Ethics Committee at the University of Melbourne (HREC #1238319.3). The data generated by this study is free and openaccess online at https://dataverse.harvard.edu/dataverse/kpeyton.

The primary outcome measure in Experiments 1–3 is “political trust” or “trust in government,” a concept widely used in survey research to capture the generalized attitude individuals have toward the trustworthiness of government. Experiments 1–3 used two measures of political trust: a four-item Likert scale that has been found to be internally reliable in previous research [19], and a single-item measure from the American National Election Study (ANES), “How much of the time do you think you can trust the government in Washington to do what is right?”. For the Likert Scale, respondents are asked to indicate their agreement with each of the following statements on a 6 point scale from “Strongly Disagree” to “Strongly Agree”:

1. We generally cannot trust politicians.
2. People in government are too often interested in looking after themselves.
3. Government is run by a few big interests who look after their own interests.
4. A lot of politicians are corrupt.

We conducted analyses to confirm the reliability of the 4-item political trust measure and found it satisfied canonical psychometric criteria for scalability (Cronbach’s alpha > 0.90 in all studies). We note however that while the 4-item measure is intended to measure political trust several of these items could tap attitudes towards politicians’ integrity and honesty, thereby potentially conflating trust with political probity. As the ANES trust measure does not suffer from this problem, it provides a more compelling test of the causal link between probity and trust. Nonetheless, we report analyses of both measures and, as reported in detail below, we find that they yield similar results. The SI provides additional details, including the full text of all articles, further elaboration of the design and sample characteristics for each experiment, and analyses for a post-treatment manipulation check using the 5-item political probity scale.

### Experiments 1–2

After completing a short background questionnaire, subjects were assigned to one of three conditions: *Control*, *Corrupt*, or *Honest*. In *Control*, subjects read a *New York Times* article about celebrities Anthony Bourdain and Eric Ripert. In *Corrupt*, subjects read a putative opinion piece (“Op-Ed”) written in *The New York Times* by an Adjunct Professor of Law at the University of Chicago and former prosecutor in the FBI’s Public Integrity Section titled “Political Corruption is Rampant,” that characterized political elites (politicians and government officials) as dishonest, and argued that political corruption in the United States was widespread. In *Honest*, participants read an Op-Ed by the same author, titled “It Only Seems that Political Corruption is Rampant,” that described low levels of political corruption in the United States and the generally good character of elected officials and other government employees.

Consistent with prior scholarship, we conceptualized political probity in terms of the mass public’s judgements about the moral character of political authorities. Experiments 1–2 depart from prior studies that use real corruption scandals to study probity [20, 21] in two important respects. First, the Op-Ed treatments were designed to persuade and inform subjects about the presence (or absence) of broader corruption in politics, and were therefore portrayed to be written by a credible non-partisan source and devoid of any explicitly partisan content or references to corruption scandals (e.g. Watergate) associated with any particular political party. Second, we study the instantaneous effect of a single signal about political probity as opposed to the over-time effects that multiple incidents may have on public opinion. The advantage here is that we can isolate the impact of a particular event (reading an article about corruption) in a controlled environment to provide an experimental test of the hypothesis that signals of political probity (or its absence) affect political trust.

### Experiment 3

One potential concern with the Op-Ed treatments in Experiments 1–2 is that the psychological mechanism driving any change in political trust might be attributable to content valence, such as the “warm glow” associated with “good news,” since positive (or negative) stimuli tend to generate positive (or negative) responses [22]. Experiment 3 investigates this possibility by randomly assigning subjects to read one of three news articles about the probity of NFL players and officials. In *Corrupt*, participants read an article, titled “Corruption in the National Football League is Rampant,” which described NFL players and officials as corrupt and dishonest. In *Honest*, participants read an article, titled “It only seems that Corruption in the National Football League is Rampant,” which described low levels of corruption in the NFL and honest behaviors of NFL players and officials. In *Control*, respondents read the same article about Anthony Bourdain and Eric Ripert used Experiments 1–2. The design of Experiment 3 was otherwise equivalent to Experiments 1–2 and therefore serves as a “placebo test” for the hypothesis that trust in government might be affected by similarly valenced content about non-political corruption. If observed changes in political trust in Experiment 1–2 are simply driven by content valence then we would expect to see similar changes in Experiment 3.

## Results and discussion

We estimate the impact of the *Corrupt* and *Honest* treatments on both the single-item ANES measure of political trust and the 4-item Likert Scale using Ordinary Least Squares (OLS) regression, controlling for the background attributes of respondents to increase precision:

$$Y_{i}=a+b_{1}{Corrupt}_{i}+b_{2}{Honest}_{i}+g_{1}X_{1i}+g_{2}X_{2i}+\cdots +g_{K}X_{Ki}+u_{i}$$

where *Y*_*i*_ denotes subject *i*’s reported political trust, *Corrupt*_*i*_ is an indicator for whether individual *i* was assigned *Corrupt* or *Control*, *Honest*_*i*_ is an indicator for whether *i* was assigned *Honest* or *Control* and *u*_*i*_ represents unmeasured determinants of political trust. The key parameters of interest are *b*_1_ and *b*_2_, which represent the average treatment effects of the *Corrupt* and *Honest* treatments relative to *Control*. We control for pre-treatment covariates *X*_*Ki*_ that are correlated with political trust in order to increase the precision of estimates for *b*_1_ and *b*_2_. These include income, age, conservativism, and indicators for partisanship, race, sex, education and employment. We obtain 95% confidence intervals (CIs) and *P*-values using HC2 robust standard errors and also report the correspondening difference-in-means estimates that are obtained without covariate-adjustment. To facilitate interpretation, we report standardized effect sizes using Glass’ Delta, which scales outcomes by the standard deviation in the control group and provides a more conservative estimate than standardizing using the pooled standard deviation in the presence of treatment effect heterogeneity [23].

**Fig 1:**
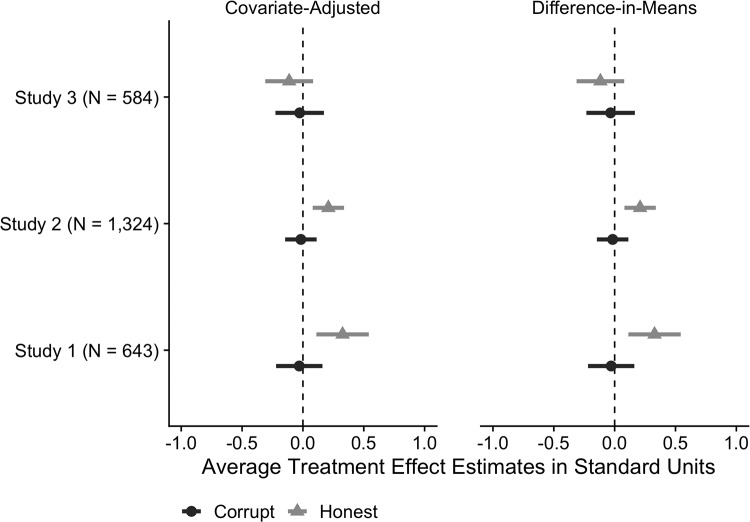
Estimated effects of *Corrupt* and *Honest* treatments on political trust (ANES Item) in Experiments 1–3.

Fig 1 summarizes the findings for the single-item ANES measure graphically with estimated treatment effects and 95% CIs from both the difference-in-means and covariate-adjusted estimators. Table 1 presents the point estimates and standard errors across all three experiments. We find that the *Honest* treatment increased political trust by approximately 0.33 standard units in Experiment 1 (*P* = 0.003) and 0.21 standard units in Experiment 2 (*P* = 0.002). The estimated effects of the *Corrupt* treatment were substantively negligible and not statistically distinguishable from zero in either Experiment 1 (-0.03, *P* = 0.76) or Experiment 2 (-0.02, *P* = 0.80). In Experiment 3, which focused on non-political corruption, the estimated effects of the *Corrupt* (-0.03, *P* = 0.79) and *Honest* (-0.11, *P* = 0.26) treatments were substantively negligible and statistically indistinguishable from zero, suggesting the increased political trust in Experiments 1–2 was driven by information about political probity rather than Op-Ed valence.

**Table 1:** Estimated treatment effects and standard errors in Experiments 1–3.

	Dependent Variable: Political Trust (ANES Item)	
	Covariate-Adjusted	Difference-in-Means
**Experiment 1**		
*Corrupt*	-0.04 (0.10)	-0.03 (0.10)
*Honest*	0.36 (0.11)***	0.33 (0.11)***
**Experiment 2**		
*Corrupt*	-0.02 (0.07)	-0.02 (0.07)
*Honest*	0.21 (0.07)***	0.21 (0.07)***
**Experiment 3**		
*Corrupt*	-0.04 (0.10)	-0.03 (0.10)
*Honest*	-0.13 (0.10)	-0.12 (0.10)

Notes: All estimates computed using ordinary least squares regression with HC2 robust standard errors. Unadjusted estimates computed without covariates. Adjusted estimates computed using linear controls for pre-treatment measures of income, age, conservativism, and indicators for partisanship, race, sex, education and employment.

Normal approximation based p-values:

*** < 0.001.

**Fig 2:**
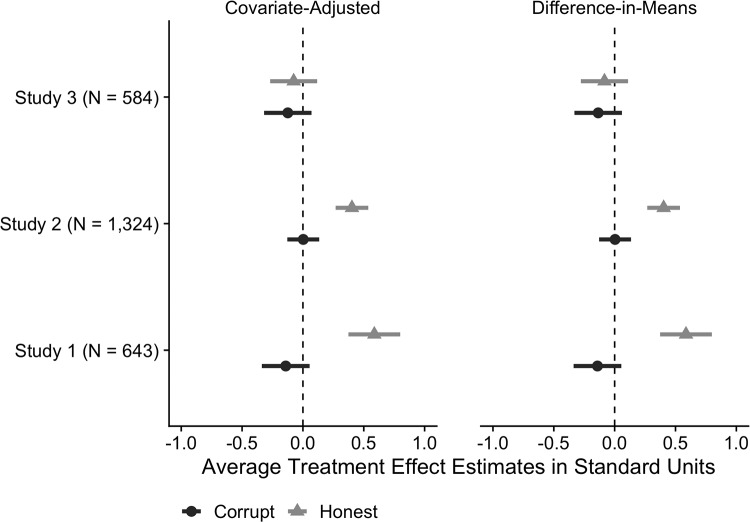
Estimated effects of *Corrupt* and *Honest* treatments on political trust (Likert Scale) in Experiments 1–3.

Fig 2 summarizes the findings for the 4-item Likert Scale graphically with estimated treatment effects and 95% CIs from both the difference-in-means and covariate-adjusted estimators. Table 2 presents the point estimates and standard errors across all three experiments. We find that the *Honest* treatment increased political trust by approximately 0.6 standard units in Experiment 1 (*P* < 0.001) and 0.40 standard units in Experiment 2 (*P* < 0.001). The estimated effects of the *Corrupt* treatment were substantively negligible and not statistically distinguishable from zero in either Experiment 1 (-0.16, *P* = 0.11) or Experiment 2 (0.01, *P* = 0.94). In Experiment 3, which focused on non-political corruption, the estimated effects of the *Corrupt* (-0.12, *P* = 0.21) and *Honest* (-0.08, *P* = 0.41) treatments were substantively negligible and statistically indistinguishable from zero, suggesting the increased political trust in Experiments 1–2 was driven by information about political probity rather than Op-Ed valence. These estimates are consistent with those presented in Fig 1 and Table 1 but subject to more uncertainty, which may be attributable to measurement error [24].

**Table 2:** Estimated treatment effects and standard errors in Experiments 1–3.

	Dependent Variable: Political Trust (Likert Scale)	
	Covariate-Adjusted	Difference-in-Means
**Experiment 1**		
*Corrupt*	-0.16 (0.10)	-0.14 (0.10)
*Honest*	0.61 (0.11)***	0.59 (0.11)***
**Experiment 2**		
*Corrupt*	0.01 (0.07)	0.00 (0.07)
*Honest*	0.40 (0.07)***	0.40 (0.07)***
**Experiment 3**		
*Corrupt*	-0.12 (0.10)	-0.14 (0.10)
*Honest*	-0.08 (0.10)	-0.08 (0.10)

Notes: All estimates computed using ordinary least squares regression with HC2 robust standard errors. Unadjusted estimates computed without covariates. Adjusted estimates computed using linear controls for pre-treatment measures of income, age, conservativism, and indicators for partisanship, race, sex, education and employment.

Normal approximation based p-values:

*** < 0.001.

We view the estimated effects of the *Honest* treatment in Experiments 1–2 as substantively important in this context. One benchmark for comparison is the “partisan gap” between Democrats and Republicans. For example, previous studies have shown that partisanship is one of the strongest drivers of political trust in the US with voters reporting higher levels of trust when their party is in power [14, 25]. Pooling across all three Experiments, the average difference between Republicans and Democrats in the *Control* group was approximately 0.20 standard units on the Likert Scale and 0.40 standard units on the single-item ANES measure, suggesting the *Honest* treatment increased political trust by an amount more than double the partisan gap on the former measure, and about the same size as the partisan gap on the latter measure.

The partisan gap in political trust between Republicans and Democrats is well documented. All else equal, contemporary Republicans express much lower levels of trust in government. The “polarized trust'' hypothesis holds that these differences help explain system-level political dysfunction and policy gridlock. A pessimistic interpretation of this phenomenon is that attitudes about trust should, like climate change, be added to the list of fundamental partisan differences. If true, it would be surprising to find the attitude changes we observe here in response to a single piece of information. However, it may simply be the case that Democrats became even more trusting of government whereas Republicans did not change at all.

**Fig 3:**
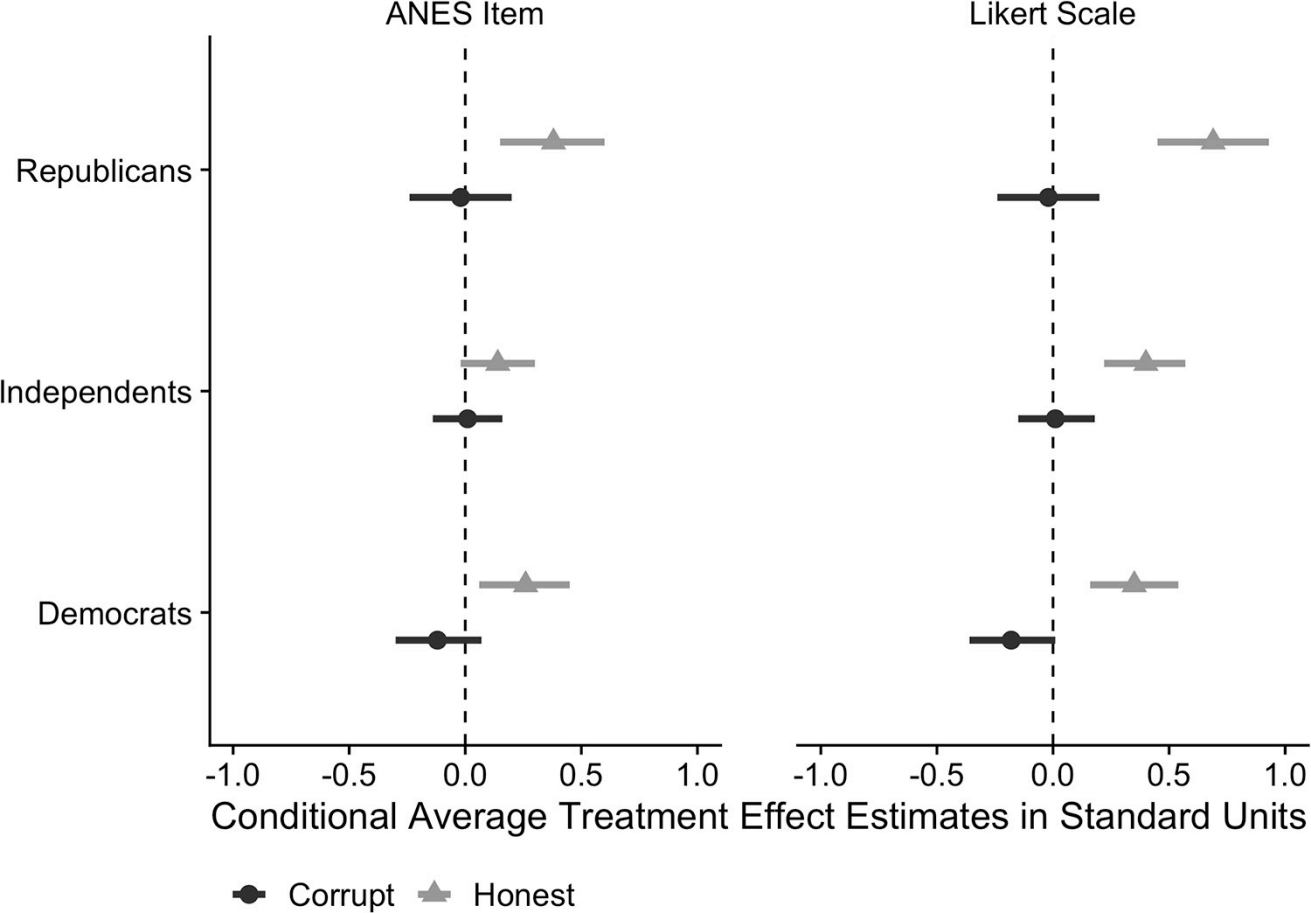
Estimated effects of *Corrupt* and *Honest* treatments by party identification and outcome measure.

All of our treatments were deliberately designed to be devoid of partisan content, and we can explore the hypothesis that partisan groups respond differently to treatment by combining the observations in Experiments 1–2 and estimating the Conditional Average Treatment Effects (CATEs) for each partisan sub-group. These results are presented in Fig 3 and Table 3 for each measures of political trust. We find that the *Honest* treatment was slightly more effective among Republicans (0.38 standard units for the ANES item and 0.69 standard units for the Likert Scale) than Independents (0.14 standard units for the ANES item and 0.40 standard units for the Likert Scale) or Democrats (0.26 standard units for the ANES item and 0.35 standard units for the Likert Scale), although these differences in CATEs are not statistically distinguishable across partisan groups. This analysis demonstrates that even though Republicans are more cynical about government than Democrats, their attitudes are nevertheless responsive to increases in political probity, suggesting that partisan differences in political trust are more malleable than suggested by prior work [14]. This finding is noteworthy since these experiments were conducted in 2014 under Democratic President Barack Obama, when Republicans were significantly less trusting of government than Democrats.

**Table 3:** Estimated treatment effects and standard errors by party identification and outcome measure.

	Dependent Variable: Political Trust	
	ANES Item	Likert Scale
**Republicans**		
*Corrupt*	-0.02 (0.11)	-0.02 (0.11)
*Honest*	0.38 (0.11)*	0.69 (0.12)*
**Independents**		
*Corrupt*	0.01 (0.08)	0.01 (0.09)
*Honest*	0.14 (0.08)	0.40 (0.09)*
**Democrats**		
*Corrupt*	-0.12 (0.10)	-0.18 (0.09)
*Honest*	0.26 (0.10)*	0.35 (0.10)*

Notes: Covariate-adjusted estimates computed using ordinary least squares regression with HC2 robust standard errors. Separate regressions estimated for each outcome measures and partisan sub-group (Republicans: 480; Independents: 796; Democrats: 691) using linear controls for pre-treatment measures of income, age, conservativism, indicators for race, sex, education and employment, and a fixed effect for study population (Experiment 1 or 2).

Normal approximation based p-values:

* < 0.05.

The findings reported here demonstrate that political probity plays a distinct role in shaping political trust. These findings demonstrate that probity is an important cause of political trust in the United States, as suggested by prior observational studies. One implication is that trust in government may be a reflection of the public’s belief about the presence (or absence) of corruption, rather than (or in addition to), prevailing economic conditions and partisan disagreements about the role of government. A related implication is that trust is subject to experience based revision, rathen than a fixed world view with purely ideological or partisan origins.

### Limitations

Although we found that signaling political probity via information about a lack of corruption in government clearly increases political trust, we were unable to reject the null hypothesis of no average difference in political trust between subjects in the *Corrupt* and *Control* conditions. We consider two plausible explanations for this asymmetry. First, this may be attributable to measurement error due to censoring. For example, if values less than 4 cannot be recorded by the survey instrument (a 4-item Likert scale) but the negative trust treatment nevertheless reduces the latent attitude of interest then OLS estimates will be biased toward zero. Consistent with this possibility, baseline levels of political trust are at the lowest end of the survey instrument: across all studies, over 80% of the subjects in *Control* gave negative ratings on all four items used to construct the political trust scale, and about 30% gave the lowest possible rating. These low levels of trust in government are consistent with the numerous surveys of public opinion showing Americans report unusually low levels of political trust, especially relative to other developed democracies [26]. We explore this in the SI and find that estimates from a Tobit regression that accounts for this censoring are similar to those obtained from OLS.

A second possibility, predicted by Bayesian models of rational learning [27, 28], is that although individuals view information that conforms with their pre-existing views (e.g. that most politicians are corrupt) as persuasive, the strength of belief change is proportional to the degree that new evidence deviates from prior beliefs (e.g. that most politicians are honest), provided it comes from a credible source. The fact that Americans have very low baseline levels of political trust suggests that additional information that conforms to those existing beliefs should be less powerful than information that challenges those beliefs. We believe this feature of the American population explains, in large part, why the effect of information exposing the corrupt behavior of political elites had comparatively little effect on political trust. Consistent with this conjecture, we also find that subjects in the *Honest* treatment were much more likely to say the article changed their views even though subjects in the *Corrupt* treatment viewed the article as more persuasive (See Supplementary Tables 1–10 in S1 Appendix).

Another caveat is that although prior experimental research has shown that Op-Eds have strong persuasive effects that persist for at least a month [18], we cannot directly asses the durability of the effects induced here since follow-up surveys were not conducted. A related experiment found televised incivility in political discourse decreased trust in government, and that these effects were detectable in follow-up surveys three weeks later, attrition and sample size constraints limited any firm concslusions about persistence at a one month follow up [29]. Although this finding is potentially complementary to what we report here, political discourse that violates social norms of politeness is a different mechanism than corruption. Longitudinal survey experiments that study the dynamics of belief updating over time could shed additional light on whether Op-Ed treatments like the *Honest* one used here also have durable effects on political trust.

Finally, as with any form of empirical social science research, we cannot rule out the possibility that some individuals may have been motivated to respond in what they perceived to be the socially desirable direction. Concerns about so-called “demand effects” may be an important threat to internal validity in lab-style experiments where, for example, undergraduate research subjects may be motivated by social image concerns to behave differently than they would were a professor not present [30]. In our setting, the potential threat to inference might be that subjects in the *Honest* treatment arm deduced the purpose of the study and subsequently altered their responses, perhaps in order to please the experimenter. However, in light of the large body of empirical research that has been conducted on this topic, the emerging consensus is that—if they exist—experimenter induced demand effects are substantively neglibile in anonymous online survey experiments [31–34]. Further, it is unclear what direction a hypothetical demand effect might run in this setting and if one did exists it is not obvious why it would bias responses away from zero in the *Honesty* treatment arm but not the *Corrupt* treatment arm.

## Conclusions

Political trust has eroded across the advanced democracies, and low trust in government is an especially chronic problem in the United States. Understanding the causes of political trust has been an important research priority since the “trust in government” question first appeared in the American National Election Studies (ANES) Survey in 1958 [16, 35]. In this article we have used randomized survey experiments to collect new data about political probity, a widely cited potential causal mechanism [6, 13–18, 35]. The causal estimates reported here support this large body of theory and observational studies that have linked probity to popular trust in government. Experiments 1 and 2 showed that exposure to information about the trustworthy behavior of political elites via Op-Eds about political corruption has a large positive effect on political trust, and the results from Experiment 3 suggest voters’ perceptions about *politicians and government*–rather than the valence of media content—plays a distinct role in shaping political trust.

Another implication is that the public’s trust in government may be more of a reflection of current beliefs about the presence (or absence) of corruption than economic conditions or fundamental partisan disagreements about the role of government. The results in Experimens 1–2 challenge the conventional view of poltical trust as “a more stable attribute of individuals, one that changes slowly and incrementally, if at all” [29]. Given that political trust is subject to experience-based revision, and not fixed world views, we expect trust would readjust in response to new information and social interactions that occur outside the lab. Changes may be stronger, for example, in response to repeated exposure to similar media content [35, 36], broader improvements in the quality of government [37], or as a result of migration to locations with different institutions and social norms [38].

Identifying the factors that cause changes in politicial trust helps shed additional light on the potential mechanisms behind the erosion of trust in government in many advanced democracies. More than a decade ago, reviews of the large body of survey research on political trust concluded that experimental research was necessary to advance this research agenda [34], yet few experimental studies have been published since then. Those that have are primarily focused on identifying factors that *decrease* political trust. One of the earliest such studies—a lab experiment on political discourse—showed that televised public incivility decreased political trust but polite disagreements about differences of opinion did not, suggesting the meteoric rise of political news in the United States is an important causal factor of declining trust in government [28]. Another lab-style experiment on Australian undergraduates found that newpaper content describing politicians as ‘dishonest’, ‘decieving’ and ‘sly’ also reduced their trust in government [20]. Related studies have also found that exposing political corruption has demobilizing effects on political participation and voter turnout [39–41]. More recently, a series of field experiments found that publicizing political scandals via newspaper articles has strong and durable effects on political trust [42]. The experiments reported here add to this small but growing body of published experimental research on political trust, and demonstrate that exposure to information about the probity of political elites can generate substantively large increases in political trust.

## Supporting information

S1 Appendix (PDF)
