# Supplementary material for: Political probity increases trust in government: Evidence from randomized survey experiments
Source: PLoS One. 2020 Feb 24;15(2):e0225818. doi: 10.1371/journal.pone.0225818 (PMC7039441; doi:10.1371/journal.pone.0225818)
Supplement: S1 Appendix — (PDF) [file pone.0225818.s001.pdf]

Supplementary Appendix for "Political Probity Increases Trust in Government: Evidence from Randomized Survey Experiments"

Contents

List of Figures 2

List of Tables 2

1 Design 3

1.1 Study 1 4

1.1.1 Treatment 1: Honest politicians 4

1.1.2 Treatment 2: Corrupt politicians 6

1.1.3 Treatment 3: Control 8

1.1.4 Sample 10

1.2 Experiment 2 10

1.2.1 Treatment 1: Honest politicians 10

1.2.2 Treatment 2: Corrupt politicians 11

1.2.3 Treatment 3: Control 11

1.2.4 Sample 11

1.3 Experiment 3 12

1.3.1 Treatment 1: Honest NFL 12

1.3.2 Treatment 2: Corrupt NFL 14

1.3.3 Treatment 3: Control 15

1.3.4 Sample 16

1.4 Covariate balance 16

2 Measurement 17

2.1 ANES Measure of Political Trust 18

2.2 Manipulation checks 18

2.3 Measurement error due to censoring? 21

Bibliography 23

3 Appendix A: Qualtrics Survey Instruments

List of Figures

S1 Fig. Experiment flow diagram . . . 3
S2 Fig. De-brief prompt . . . 4
S3 Fig. Randomization Inference for Covariate Balance in Experiments 1-3 17
S4 Fig. Average Treatment Effect Estimates on Political Probity (in Standard Units) . . . 21

List of Tables

S1 Table. Allocation of subjects across experimental conditions in Experiment 1 . . . 10
S2 Table. Descriptive statistics/selected variables for Experiment 1 . . . 10
S3 Table. Allocation of subjects across experimental conditions in Experiment 2 . . . 11
S4 Table. Descriptive statistics/selected variables for Experiment 2 . . . 11
S5 Table. Allocation of subjects across experimental conditions in Experiment 3 . . . 16
S6 Table. Descriptive statistics/selected variables for Experiment 3 . . . 16
S7 Table. Internal reliability of Political Trust instrument . . . 18
S8 Table. Group means for questions after articles . . . 19
S9 Table. Internal reliability of Political Probity instrument . . . 20
S10 Table. Average Treatment Effect Estimates on Political Probity (in Standard Units) . . . 21
S11 Table. Estimated Effect of Corrupt Treatment on Political Trust (with 95% confidence intervals) . . . 22

1 Design

S1 Fig illustrates the basic design of all three studies reported in the paper. Pre-treatment questions included standard demographic measures and party identification. In studies 1 and 3, participants were allocated to one of the three treatments with equal probability according to simple random assignment. Experiment 2 deviated from the simple design and used block random assignment by party identification to ensure balance in partisanship across treatment conditions. The treatments assigned in Experiment 1 and 2 were identical. Treatment 1 and 2 in Experiment 3 were different. Complete details about treatments for each study appear in this document.

S1 Fig. Experiment flow diagram

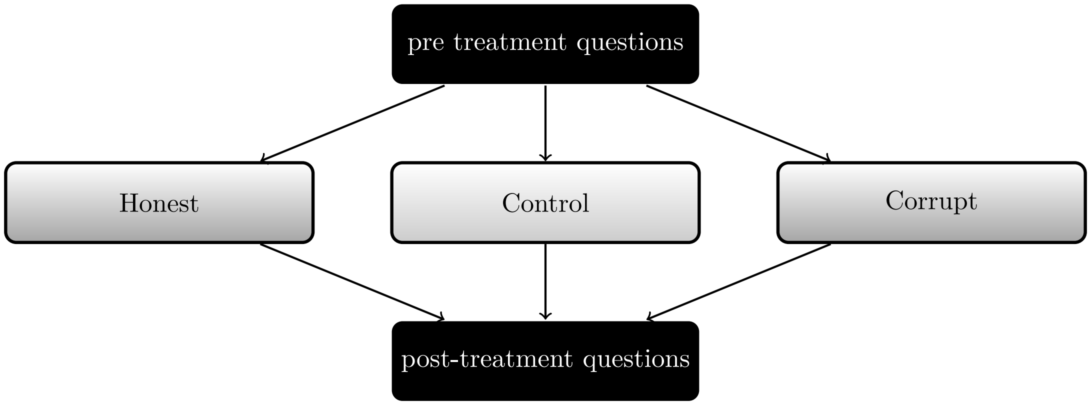

All studies were conducted using Qualtrics survey software. Additional details about the sample recruited for each study are provided in this document. All studies assigned putative *New York Times* opinion pieces written by an adjunct professor of law at the University of Chicago and former Chief Prosecutor in the United States Department of Justice's Public Integrity Section. All articles were 503 words in length and are presented here in full. The fictional articles were necessarily deceptive. This deception was approved by our Human Ethics committee conditional on a de-brief at the end of the survey with an option for participants to remove their data. S2 Fig shows the prompt. All subjects that elected to have their data removed were excluded from the analysis. There is no evidence that these decisions were a function of treatment assignment in any of our studies (more details below).

S2 Fig. De-brief prompt

Thank-you for participating in this study.

At the beginning of the study we only gave you a brief idea of the experiment's purpose. Sometimes when we are studying how people think about social issues (as in this experiment) we don't give people a full description of what we are studying. That way we are able to get natural responses. As such, there are a few things about this experiment that we would like to explain.

The true purpose of this study is to investigate how people's political attitudes are affected by reading newspaper articles about political corruption. As such, some participants were shown a news story that highlighted instances of political corruption. Other participants read a story that highlighted how political corruption is not as common as people often think. Other participants still read an article unrelated to political corruption. All of the articles were fictional.

If you feel concerned or uncomfortable about the fact that you were deceived, you may tell us to withdraw your data from the sample by checking the box at the bottom of this page. Remember that your results are confidential to the researchers, and that all results are published anonymously as group data.

We will be running this experiment for some time. We would really appreciate it if you would not talk to anyone about the study. Sometimes if people know what the study is about, that knowledge will affect their responses even when they don't mean for it to, and then the data are not valid. If you are asked about what you did in the study, please merely say that you had to read a news article and respond to some questions about it.

Thank you again for your valuable contribution to this research!

Please check the below box only if you want to withdraw your data. Otherwise, do not check the box.

☐ Having read the above, I would like to withdraw my data.

As part of standard operating procedures in obtaining consent, all participants were instructed that their responses are anonymous and ensured that their responses would not affect their payment for participation prior to entering the survey. Immediately after reading the articles, subjects were asked to reflect upon the articles and say whether they found them convincing.

Next, before any outcomes were measured, subjects were again reminded that their responses were anonymous with the prompt: "In the following sections you will be asked a number of questions without interruption. Please answer all questions honestly and remember that your responses are completely anonymous."

They also answered "manipulation check" questions about perceived political probity that were adopted from (Rose, 2014). These questions, along with the article engagement questions asked immediately after treatment are analyzed in Section 2.

1.1 Study 1

1.1.1 Treatment 1: Honest politicians

Prompt:

> In the next section you will see an opinion piece about political corruption written in The New York Times by Charles Delauney, an adjunct professor of law at

the University of Chicago and former Chief Prosecutor in the United States Department of Justice's Public Integrity Section. Please pay attention to the article as you will later be asked questions about the content.

It Only Seems That Political Corruption is Rampant

Much has been written about political trust lately. It has become very fashionable to call politicians distrustful. Americans, of course, have often been skeptical about government and politicians, but over the last decades, this skepticism has hardened. The recent increase in media coverage of political corruption seems to suggest it is rampant; however, this rise in hardened skepticism is unwarranted. Although public perceptions that politicians are corrupt have skyrocketed, political corruption has in fact decreased over time.

I led my first prosecution of political corruption in 1992 and things have certainly improved since then. Today, political corruption is at an all-time low. According to a recent study by Larry J. Sabato, director of the University of Virginia's Center for Politics, political corruption was much more common throughout the 19th and 20th centuries than today. The idea that aspiring politicians are driven to a career in politics solely for their own personal gain is nonsense. Most politicians work significantly longer hours and get paid considerably less than they would in the private sector. And they face intense and, increasingly, vindictive media scrutiny.

It is true that there are occasional corruption scandals involving politicians, but the perception of widespread malfeasance is primarily driven by media scrutiny. In contrast to countries like Italy where political scandals are the norm, we pay them so much attention in the United States precisely because they are so unusual. One occasional rotten apple, or even a handful over the past 20 years, does not spoil the barrel.

Political corruption is simply a rare event in America and although media coverage is easy to find the facts tell a different story. I worked for 25 years as a prosecutor in the Justice Department's

public integrity section, which prosecutes official corruption at all levels of government. Although resources devoted to prosecuting corrupt officials have steadily increased since the 1980s, convictions of politicians dropped nearly a quarter from 1989 to 2011. This reflects the fact that only a very few politicians engage in corruption.

The relatively small percentage of corrupt politicians in the United States is well known by scholars. A 2007 book called *The Almanac of Political Corruption, Scandals and Dirty Politics*, concluded that less than 1 percent of the nearly 12,000 people who had served in Congress had been expelled, indicted or tried for crimes. According to the book's author, Kim Long: "There's a large majority of voters who believe it's just endemic ... There's no evidence that indicates it's the case – zero." I couldn't agree more.

Political corruption has always existed and the only thing rampant is the public misconception about political corruption. The view of corruption as commonplace is not supported by the evidence. In my experience as a prosecutor the facts best represent the reality that political corruption in America is a very rare event. Although it is not reflected in public discussion and media coverage, using words like truthful, honest, sincere, loyal, and genuine to describe most politicians is not as ridiculous as it may sound.

1.1.2 Treatment 2: Corrupt politicians

Prompt:

In the next section you will see an opinion piece about political corruption written in The New York Times by Charles Delauney, an adjunct professor of law at the University of Chicago and former Chief Prosecutor in the United States Department of Justice's Public Integrity Section. Please pay attention to the article as you will later be asked questions about the content.

Political Corruption is Rampant

Much has been written about political trust of late. It has become very fashionable to call politicians distrustful. Americans, of course, have often been skeptical about government and politicians, but

over the last decades, this skepticism has hardened. This rise in hardened skepticism is certainly warranted. In the last few decades, the United States has suffered a tremendous increase in the scope and frequency of political corruption.

I led my first prosecution of political corruption in 1992 when an F.B.I. sting involving bribery and horse-racing legislation netted convictions of nearly 11 percent of the Kentucky Legislature. And since then things have only gotten worse. Just last year three members of the House of Representatives pleaded guilty to, or were convicted of crimes, more than any other year since 1981. It would seem that many aspiring politicians enter politics as a 'career' or, even worse, a racket, rather than a vocation.

The problem is becoming increasingly common. Earlier this year, former Virginia governor Bob McDonnell became the ninth governor or former governor to be charged with a crime since 2000. In 2009, House legislator William Jefferson was found with 90,000 in his basement freezer and convicted of bribery, racketeering and money laundering. A 2012 study by researchers at the University of Illinois at Chicago calculated that 31 of the approximately 100 Chicago aldermen who had served since 1973 – and four of the seven Illinois governors – had been convicted of corruption.

Stories like these are becoming increasingly commonplace and reflect the growth of political corruption in America. I worked for 25 years as a prosecutor in the Justice Department's public integrity section, which prosecutes official corruption at all levels of government. Resources devoted to prosecuting corrupt officials have steadily increased since the 1980s and the Federal Bureau of Investigation said in 2011 that it was conducting more than 2,000 corruption inquiries and had secured more than 900 convictions in fiscal 2010.

The relatively large percentage of corrupt politicians in the United States is well known by scholars. According to Kim Long who published *The Almanac of Political Corruption, Scandals and Dirty*

Politics, political corruption in American predates the origins of the republic. According to Long, corruption during the British administration of the colonies was "routine and not necessarily illegal. That set the stage for an underlying culture of corruption and patronage that ensued after the revolution." I couldn't agree more.

Although political corruption has always been a problem in America it has become so commonplace that many new acts of corruption are not even reported by the media. I would like to say that my experience working as a prosecutor in the Department of Justice made me believe political corruption was under control in the United States. In the far distant past, I did believe this. Today, however, the sheer volume of corruption cases is overwhelming the teams of prosecutors assigned to them. I have to admit that the general view of politicians as distrustful and corrupt is, sadly, a very accurate one.

1.1.3 Treatment 3: Control

Prompt:

In the next section you will see an article written in *The New York Times* about Chefs Anthony Bourdain and Eric Ripert. Please pay attention to the article as you will later be asked questions about the content.

Boisson Buddies: Anthony Bourdain and Eric Ripert

On paper they couldn't be more different–one is a refined French chef with four–stars from *The New York Times*, three from the Michelin Guide, and a number of awards from the James Beard Foundation including Top Chef in New York City and Outstanding Chef in the United States; the other is a New York City-born, five-time Emmy-nominated, world-traveling culinary renegade whose first novel, *Bone in the Throat*, is being adapted for the big screen.

Yet Le Bernardin's Eric Ripert and best-selling author and television host Anthony Bourdain go together like moules and frites. Whereas Ripert, his wife, Sandra, and their son, Adrien, have been summering in the Hampton's for more than a decade, this season

is the first for Bourdain and his wife, Ottavia, and their daughter, Ariane. But it is a wonderful respite for Bourdain, who spent 260 days last year traveling for his two Travel Channel series, *No Reservations* and *The Layover* and will launch his graphic novel *Get Jiro!* in the fall.

It was in the least likeliest of ways that these two toques came together. When Bourdain turned the heat up on the culinary industry in his 2001 breakout book *Kitchen Confidential: Adventures in the Culinary Underbelly*, he had many complementary passages about Ripert and Le Bernardin.

“Seventy-five percent of the industry was saying, ‘it’s scandalous’ and ‘this guy is a disgrace.’ Then part of the industry was saying, ‘he’s genius,”’ remembers Ripert. “I called him and said, ‘I read your book, and I would love to know you. Would you come for lunch?’ That was the first time I met Anthony, and we have been friends ever since.”

“When Eric called, the book was doing really well, but I was still working every day at [Brasserie] Les Halles, convinced, quite certain that I should keep my day job and that there was no way that I would be able to support myself or count on writing as an income stream of any kind” says Bourdaindain. “I was absolutely floored that a chef who I respected that much from a restaurant that I never could have been able to afford would call me up and invite me to lunch. When I had the opportunity to get really good at my craft, I chose not to and went the other way. I’m sure in many ways it has been trying to maintain and protect the reputation of an establishment like Le Bernardin and have a friend like me who is likely to put his foot in his mouth every five minutes. It speaks well of Eric’s character.”

Even stranger, a filmmaker shot me as I’m leaving the lunch, and I’m devastated, just standing there practically in tears. I’d had this amazing meal, and I really saw the road not taken. I had made some very basic decisions about my career, either knowingly or in

a calculated way. When I had the opportunity to get really good at my craft, I chose not to and went the other way.

1.1.4 Sample

692 subjects were recruited via MTurk and 20 respondents were excluded for having the same IP Address. Of the remaining 672 subjects, 29 asked to have their data removed from the experiment after learning about deception in the debrief. These responses were removed in accordance with IRB requirements. An F-test from a linear regression of removal on treatment assignment confirmed that assignment was not predictive of the removal request (p-value = 0.78). The final sample size was 643. S1 Table shows the allocation of the remaining subjects across the three conditions:

S1 Table. Allocation of subjects across experimental conditions in Experiment 1

	Control	Corrupt	Honest	Total
N	217	215	211	643

S2 Table shows sample characteristics.

S2 Table. Descriptive statistics/selected variables for Experiment 1

Statistic	N	Mean	St. Dev.	Min	Max
Female	642	0.46	0.50	0.00	1.00
Age	642	35.36	11.83	19.00	80.00
College degree	643	0.49	0.50	0.00	1.00
Asian	642	0.05	0.22	0.00	1.00
Black	642	0.08	0.28	0.00	1.00
Hispanic	642	0.04	0.20	0.00	1.00
White	642	0.80	0.40	0.00	1.00
Democrat	642	0.44	0.50	0.00	1.00
Republican	642	0.18	0.39	0.00	1.00

1.2 Experiment 2

1.2.1 Treatment 1: Honest politicians

Identical to Treatment 1 in Experiment 1.

1.2.2 Treatment 2: Corrupt politicians

Identical to Treatment 2 in Experiment 1.

1.2.3 Treatment 3: Control

Identical to Treatment 2 in Experiment 1.

1.2.4 Sample

1474 subjects were recruited via Qualtrics Panels and 1452 remained after excluding duplicate IP Addresses. completed the experiment. Of these, 128 asked to have their data removed from the experiment after learning about deception. An F-test from a linear regression of removal on treatment assignment confirmed that assignment was not predictive of the removal request (p-value = 0.38). The final sample size was 1324. S3 Table shows the allocation of subjects across experimental conditions.

S3 Table. Allocation of subjects across experimental conditions in Experiment 2

	Control	Corrupt	Honest	Total
N	443	430	451	1324

S4 Table shows sample characteristics.

S4 Table. Descriptive statistics/selected variables for Experiment 2

Statistic	N	Mean	St. Dev.	Min	Max
Female	1324	0.49	0.50	0.00	1.00
Age	1321	43.39	16.33	18.00	88.00
College degree	1324	0.41	0.49	0.00	1.00
Asian	1324	0.05	0.22	0.00	1.00
Black	1324	0.13	0.33	0.00	1.00
Hispanic	1324	0.17	0.37	0.00	1.00
White	1324	0.64	0.48	0.00	1.00
Democrat	1324	0.31	0.46	0.00	1.00
Republican	1324	0.27	0.45	0.00	1.00

This was a US General population sample. We requested the following quotas in advance:

1. Age: Atleast 86% of sample less than 65 years old

2. Sex: 50/50 balance

3. Race: 63% of the population should be white, 13% should be black and 17% should be Hispanic.

4. Education: 85% High School or higher. At least 28% bachelors degree or higher.

5. Party identification: 42% should be Independent; 25% Republican; 31% Democrat

We were particularly interested in obtaining a more representative sample by race and party identification. It is well known that MTurk workers tend to skew liberal and that both Black and Hispanic races are underrepresented (see Berinsky, Huber and Lenz, 2012). The party identification quotas were chosen in light of recent Gallup polls showing the increasing proportion of self-identified Independents in the United States[1]. The race quotas were chosen based on 2013 census estimates[2]. All quotas were met, so this sample is a good approximation to a nationally representative sample of Americas on these observables.

1.3 Experiment 3

1.3.1 Treatment 1: Honest NFL

Prompt:

> In the next section you will see an opinion piece about corruption in the National Football League (NFL) written in *The New York Times* by Charles Delauney, an adjunct professor of law at the University of Chicago and former Chief Prosecutor in the United States Department of Justice's Public Integrity Section. Please pay attention to the article as you will later be asked questions about the content.

> **It only seems that Corruption in the National Football League is rampant.**
>
> Much has been written about trust and the National Football League (NFL) recently and it has become very fashionable to call NFL players distrustful. Prominent scandals have always been part of American sports leagues. Rumors that the White Sox threw the 1919 World Series resulted in a national crisis as owners of Major League Baseball (MLB) teams worried the game lost the public trust. This lack of trust seems to have hit the NFL in recent years, yet the integrity of NFL officials and players is better than ever.
>
> According to Larry Sabato, director of the NFL Arrests Database at the University of Virginia, poor behavior by players was much

[1] see: http://www.gallup.com/poll/180440/new-record-political-independents.aspx

[2] see http://quickfacts.census.gov/qfd/states/00000.html

more common 50 years ago than today. Despite what media reports claim, most major league players are responsible citizens. Although public perceptions that the NFL and its players are corrupt have skyrocketed, the data suggest the opposite.

The endless media stories about the NFL simply reflect the fact that sports players are under constant media scrutiny. There is no evidence to suggest anything unusual about NFL players. On the contrary, a report by *The New York Times* showed arrest rates among players are extremely low compared to national averages. Based on data from the Bureau of Justice Statistics Report and the USA Today NFL Arrests Database, the arrest rate for players is only 13% of the national average for men of comparable age.

The real trend is that players are swiftly punished for their behavior off the field. For example, Viking's Adrian Peterson was recently suspended without pay until April 2015 following allegations of whipping his child with a tree branch. Suspensions of this length prior to conviction are unprecedented. Consider the 1998 case of St. Louis Rams' Leonard Little, who crashed into and killed Susan Gutweiler. He had a blood alcohol level over twice the legal limit and received four years probation and community service. He was only suspended for eight games and played for the Rams until 2009. Punishment has become more severe since then and violent behavior is down as a consequence.

Player misconduct is increasingly rare in both occurrence and severity. In 1999 Alfred Blumstein, a leading criminologist, compared rates of criminal violence among NFL players to the general population and found that the annual rate of assault and domestic violence by NFL players was less than half the national average. The recent *The New York Times* report shows incidents of player misconduct have decreased over time. Blumstein and colleagues have repeated shown that NFL players are less violent than the general population and that player conduct has not deteriorated over time.

I couldn't agree more. It seems that the only thing rampant is the

public perception that NFL players are criminals. In my experience as a prosecutor the statistics cited above represent the reality that bad behavior by NFL players and officials is very rare. Although not reflected in public discussion and media coverage, words like truthful, honest, sincere, loyal, and genuine describe most players and NFL officials.

1.3.2 Treatment 2: Corrupt NFL

Prompt:

> In the next section you will see an opinion piece about corruption in the National Football League (NFL) written in *The New York Times* by Charles Delauney, an adjunct professor of law at the University of Chicago and former Chief Prosecutor in the United States Department of Justice's Public Integrity Section. Please pay attention to the article as you will later be asked questions about the content.

Corruption in the National Football League is rampant.

Much has been written about trust and the National Football League (NFL) recently and it has become very fashionable to call NFL players distrustful. Prominent scandals have always been part of American sports leagues. Rumors that the White Sox threw the 1919 World Series resulted in a national crisis as owners of Major League Baseball (MLB) teams worried the game lost the public trust. This lack of trust seems to have hit the NFL in recent years and the integrity of NFL officials and players is worse than ever.

Today we see cases of bad behavior by NFL players and officials in increasing scope and frequency. For example, the NFL recently suspended Baltimore Ravens player Ray Rice after he was indicted for knocking his wife unconscious in a New Jersey casino. Video footage released approximately five months later showed Rice knocking out his wife and dragging her unconscious body from an elevator. Some journalists speculated NFL commissioner Goodell attempted to cover up whether the NFL knew about the video and the National Organization for Women called for his resignation.

This case highlights a widespread problem. According to a report by The New York Times, domestic violence in the NFL is extraor-

dinarily high. Based on data from the Bureau of Justice Statistics Report and the USA Today NFL Arrests Database, the arrest rate for domestic violence among NFL players is significantly higher than men of comparable age and income.

Players are also frequently arrested for driving under the influence (DUI), assault and weapons charges yet typically avoid serious punishment. In 1998, Leonard Little of the St. Louis Rams crashed into and killed Susan Gutweiler. He had a blood alcohol level over twice the legal limit and received four years probation and 1000 hours of community service.

Little was suspended for eight games and arrested again in 2004 for DUI. He received two years probation and continued to play for the Rams until 2009. The NFL does not take player misconduct seriously at it never has. The unscrupulous reputation players have earned is well deserved and NFL corruption is at an all time high.

Incidents of player misconduct are commonplace and fostered by the NFL's disregard for standards of decency and a culture of corruption. As Pulitzer Prize winning sportswriter Thomas Boswell observed: "The NFL is the league where you hold your breath week to week, almost day to day, to find out what crime, what betrayal of trust, what warped values for the young the sport can become identified with next."

I couldn't agree more. The public perception that many NFL players are criminals is correct. Corruption and misconduct has become so commonplace that many new acts are not even reported by the media. In my experience as a prosecutor I would like to say the problem is exaggerated. In the far distant past I did believe this. Today, however, I have to admit that the general view of the NFL and its players as distrustful and corrupt is, sadly, a very accurate one.

1.3.3 Treatment 3: Control

Treatment 3 in Experiment 3 was identical to Treatment 3 from Studies 1 and 2.

1.3.4 Sample

624 subjects were recruited using MTurk and 612 remained after excluding duplicate IP Addresses. Of those remaining, 28 asked to have their data removed from the experiment after learning about deception in the debrief. An F-test from a linear regression of removal on treatment assignment confirmed that assignment was not predictive of the removal request (p-value = 0.35). The final sample size was 584.

S5 Table shows other demographic characteristics.

S5 Table. Allocation of subjects across experimental conditions in Experiment 3

	Control	Corrupt	Honest	Total
N	195	194	195	584

S6 Table. Descriptive statistics/selected variables for Experiment 3

Statistic	N	Mean	St. Dev.	Min	Max
Female	584	0.48	0.50	0.00	1.00
Age	584	33.46	11.16	18.00	84.00
College degree	584	0.47	0.50	0.00	1.00
Asian	584	0.09	0.28	0.00	1.00
Black	584	0.07	0.26	0.00	1.00
Hispanic	584	0.07	0.25	0.00	1.00
White	584	0.74	0.44	0.00	1.00
Democrat	584	0.46	0.50	0.00	1.00
Republican	584	0.15	0.35	0.00	1.00

1.4 Covariate balance

Randomization inference is used to asses covariate imbalance across treatment arms (see Gerber and Green, 2012, Chapter 4 for a textbook treatment) using the `ri2` package in `R` (Coppock, 2018). S3 Fig plots a histogram of the observed F-statistic (vertical red line), and the null distribution of F-statistics, from a regression of treatment assignment on covariates, across each experiment. In Experiment 1, approximately 13% of the simulated F-statistics were larger than the observed F-statistic of 1.37 (P-value of 0.13). In Experiment 2, approximately 21% of the simulated F-statistics were larger than the observed F-statistic of 1.14 (P-value of 0.21). In Experiment 3, approximately 94% of the simulated F-statistics were larger than the observed F-statistic of 0.56 (P-value of 0.94). Thus, the null hypothesis

that no covariates have any effect on treatment assignment, as implied by the experimental design, is not rejected for Experiments 1-3.

S3 Fig. Randomization Inference for Covariate Balance in Experiments 1-3

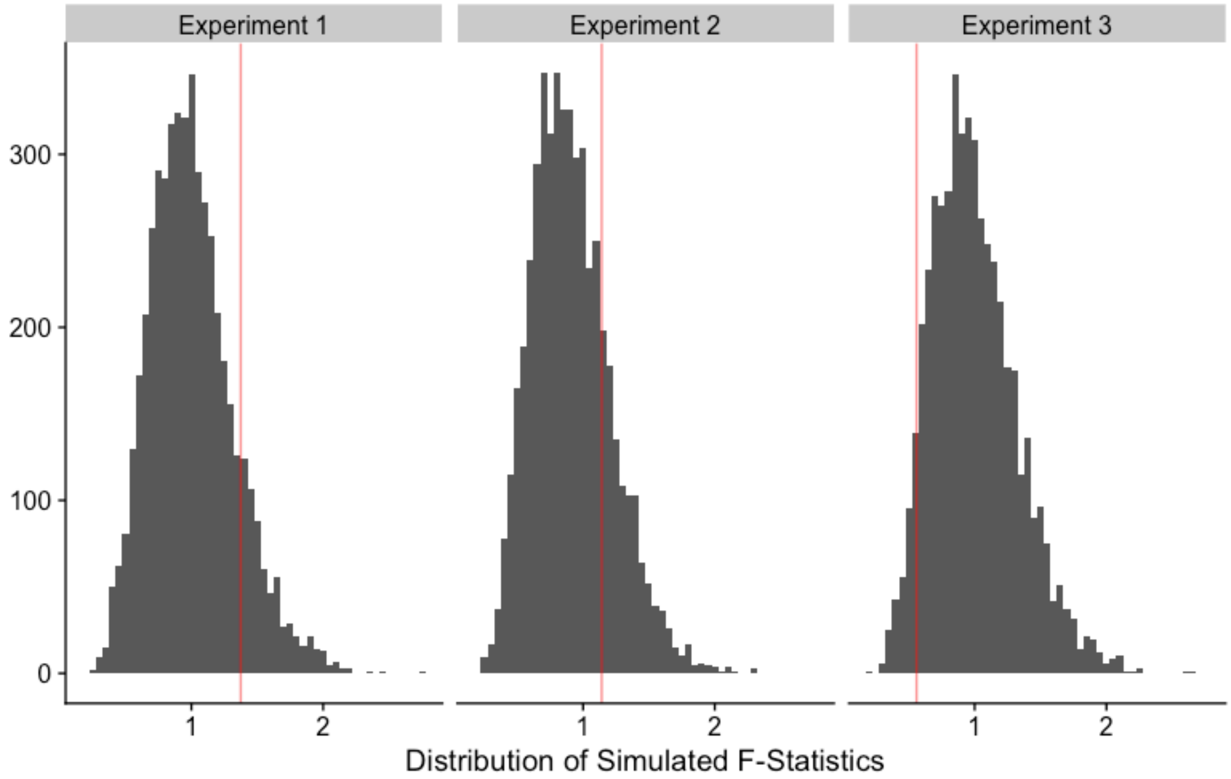

Notes: The vertical red line denotes the observed F-statistics of (1.37, 1.14, 0.56). The randomization inference P-values are 0.13, 0.21 and 0.94. The test is based on 5,000 simulations under the null hypothesis that no covariates have any effect on treatment assignment, as implied by random assignment.

2 Measurement

In all three experiments, our measures of political trust asked respondents to indicate their agreement with each of the following statements on a 6 point scale from "Strongly Disagree" to "Strongly Agree":

1. We generally cannot trust politicians.
2. People in government are too often interested in looking after themselves.
3. Government is run by a few big interests who look after their own interests.
4. A lot of politicians are corrupt.

Our primary outcome measure is a Likert scale constructed by summing these 4 items. We then standardize using Glass's delta so that effect sizes can be interpreted in standard units. The Omega statistic for internal consistency (Dunn, Baguley and Brunsden, 2013) and Cronbach's alpha, calculated using the MBESS packaged in `R` (Kelley and Lai, 2012), are reported in Table S7 with 95% confidence intervals.

S7 Table. Internal reliability of Political Trust instrument

	Statistic	
	Alpha	Omega
Experiment 1	0.90 [0.88, 0.92]	0.90 [0.87, 0.93]
Experiment 2	0.91 [0.90, 0.91]	0.91 [0.89, 0.93]
Experiment 3	0.92 [0.90, 0.94]	0.92 [0.90, 0.94]

Note: estimates are restricted to subjects in control condition across all experiments.

2.1 ANES Measure of Political Trust

In the manuscript, we also examine whether treatment effects were robust to using an alternative, single item, measure of political trust that is typically used in public opinion surveys (e.g. Gallup, Pew, ANES): "How much of the time do you think you can trust the government in Washington to do what is right?". In Experiment 1, responses were recorded using the three point scale: just about always (1), most of the time (2), only some of the time (3) used in the ANES prior to 2012. In Experiments 2-3, responses were recorded using a 5-point scale: "Always" (1), "Most of the time" (2), "About half the time" (3), "Some of the time" (4), "never" (5). We re-scaled the measure so that higher values correspond to more trust.

2.2 Manipulation checks

Immediately following the assigned article subjects were asked the following two questions:

1. **Persuasive**: How persuasive would you characterize the article you just read? [very unpersuasive (1) to very persuasive (5)]

2. **Changed**: Would it be fair to say the article you just read changed your views about the topic discussed? [Yes (1) or No (0)]

The results are presented in S8 Table . Across all three experiments, the Honest treatment was significantly more likely to have changed people's views (relative to control) according to the binary measure of self-reported view change. The Corrupt treatment, however, was deemed more persuasive by subjects than the Honest treatment (again comparing against the Control) across all three studies.

Focusing on the first two studies about political probity, subjects were more likely to report having their views changed by the Honest treatment, but also found the Corrupt treatment more persuasive. We speculate that, consistent with prior research (e.g. Gerber and Green, 1998; Bullock, 2009), subjects may be more likely to view information that conforms with their pre-existing views (i.e. that political authorities are mostly corrupt) to be more persuasive, but are more likely to update in the direction of new evidence that deviates most from their prior beliefs (i.e. that most political authorities are honest).

S8 Table. Group means for questions after articles

Measure	Study	Corrupt	Control	Honest
Changed	Study 1	0.19 (0.03)	0.18 (0.03)	0.37 (0.03)
Changed	Study 2	0.37 (0.02)	0.32 (0.02)	0.39 (0.02)
Changed	Study 3	0.26 (0.03)	0.21 (0.03)	0.45 (0.04)
Persuasive	Study 1	4.25 (0.06)	3.22 (0.06)	3.41 (0.08)
Persuasive	Study 2	4.08 (0.04)	3.47 (0.04)	3.40 (0.05)
Persuasive	Study 3	3.95 (0.07)	3.27 (0.07)	3.73 (0.07)

We also measured "probity perceptions" after exposure to treatment using 5 questions adopted from Rose (2014):

1. Overall, how would you rate the standards of conduct of public office holders in the USA? [very low (1) to very high (5)]

2. And how do you think standards of public office holders in the USA today compare with a few years ago? [Got a lot worse (1) to Improved a lot (5)]

3. How confident do you feel that the authorities in the USA are committed to improving standards in public life? [Not at all confident (1) to very confident (5)]

4. And how confident do you feel that the authorities will generally uncover wrongdoing by people in public life? [Not at all confident (1) to very confident (5)]

5. And when people in public life are caught doing wrong, how confident do you feel that the authorities will punish them? [Not at all confident (1) to very confident (5)]

We construct a Probity Index by summing these 5 items, and then standardize using Glass's delta so that effect sizes can be interpreted in standard units. The Omega statistic for internal consistency (Dunn, Baguley and Brunsden, 2013) and Cronbach's alpha are reported in Table S9 with 95% confidence intervals.

S9 Table. Internal reliability of Political Probity instrument

	Statistic	
	Alpha	Omega
Experiment 1	0.81 [0.77, 0.85]	0.82 [0.77, 0.86]
Experiment 2	0.86 [0.84, 0.88]	0.86 [0.84, 0.89]
Experiment 3	0.81 [0.77, 0.85]	0.81 [0.77, 0.86]

Note: estimates are restricted to subjects in control condition across all experiments.

As expected, this index is strongly correlated with the political trust index ($\rho = 0.53$) and the single item ANES measure ($\rho = 0.52$) among participants in the control condition (pooling across all three studies). Perceptions of political probity are also very low among control respondents. Across all three studies, the median response to each item in the Probity Index is 12 on an additive scale ranging from 5 to 25.

The point estimates (and 95% confidence intervals) associated with the treatment effects on political probity are displayed graphically in S4 Fig and also presented in S10 Table. As expected, the estimated effects in Experiment 3 are approximately zero. The estimated effects in Studies 1 and 2 are nearly identical to those for political trust – the positive treatment generated a substantial improvement in political probity, but the effect of the negative treatment was approximately zero.

S4 Fig. Average Treatment Effect Estimates on Political Probity (in Standard Units)

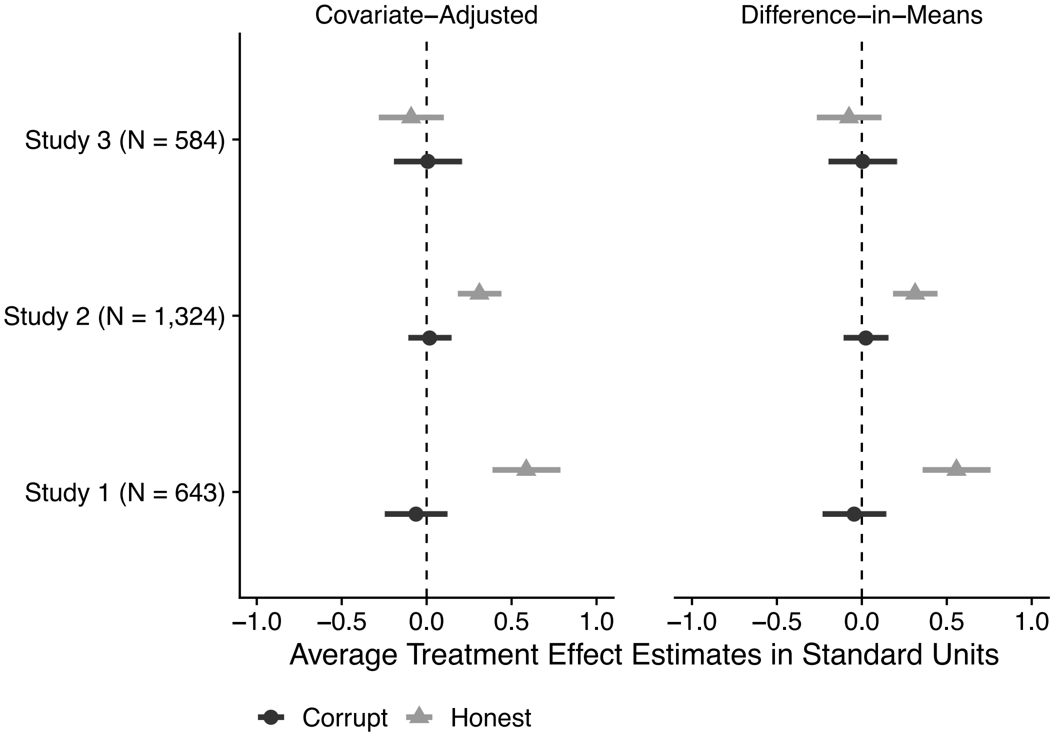

S10 Table. Average Treatment Effect Estimates on Political Probity (in Standard Units)

	Treatment	Covariate-Adjusted	Difference-in-Means
Experiment 1	Corrupt	-0.06 (0.09)	-0.04 (0.10)
Experiment 1	Honest	0.59 (0.10)*	0.56 (0.10)*
Experiment 2	Corrupt	0.02 (0.06)	0.02 (0.07)
Experiment 2	Honest	0.31 (0.07)*	0.21 (0.07)*
Experiment 3	Corrupt	0.01 (0.10)	0.01 (0.10)
Experiment 3	Honest	-0.09 (0.10)	-0.08 (0.10)

Note: stars denote statistical significance at the 5% threshold.

2.3 Measurement error due to censoring?

If decreases in political trust were actually induced in the Corrupt condition, this may have nonetheless gone undetected due to measurement error induced by censoring, sometimes called "floor effects", "left censoring" or "censoring from below". Here we further examine that possibility. The key intuition is that the existing survey instrument may be poorly suited to detect decreases in the latent attitude since the distribution of reported attitudes at baseline is highly skewed.

Let $Y_i(0)$ denote subject i's latent political trust in the untreated state where no new information about political probity is provided (the "Control" condition in our studies), and let $Y_i(1)$ denote her trust in the treated state where information about the absence of political probity is provided (the "Corrupt" condition in our studies). For simplicity, suppose the individual level treatment effect is constant and negative for all units, i.e. $\tau_i < 0 \ \ \forall\, i$, so that $Y_i(1) = Y_i(0) + \tau$. The censoring problem here can be summarized[3] as

$$Y_i^{Obs} = \begin{cases} Y_i^* & \text{if } Y_i^* > c \\ c & \text{if } Y_i^* \leq c. \end{cases}$$

Where Y_i^{Obs} denotes the observed vector of responses, c is some censoring cutpoint, and Y_i^* is the (uncensored) vector of responses that we do not observe. This unobserved response vector is connected to potential outcomes via

$$Y_i^* = Y_i(1) + [Y_i(1) - Y_i(0)] \cdot D_i = Y_i(0) + \tau D_i$$

where D_i is a binary treatment indicator. However, in the presence of "floor effects" $Y_i^{Obs} := \max\{Y_i^*, c\}$ and although τ, the causal effect of D on Y^*, is the target parameter, the difference in means estimator (regressing Y^{Obs} on D) is biased and inconsistent. If many observations are censored this bias can be quite large. The proof is straightforward and under these circumstances, a consistent estimator for τ is the well-known Tobit regression (see Amemiya, 1985; Davidson and MacKinnon, 2004, for textbook treatments).

S11 Table presents the estimated effect of the corrupt treatment on the political trust index (which has a lower bound of 4 so $c = 4$) obtained by regressing the outcome on a binary treatment indicator (1 for Corrupt and 0 for Control). As expected, estimates obtained from the Tobit estimator are lower in both studies. Compared to the OLS estimates, however, the differences are trivial.

S11 Table. Estimated Effect of Corrupt Treatment on Political Trust (with 95% confidence intervals)

	Tobit	OLS
Experiment 1	-0.80 [-1.81, 0.21]	-0.55 [-1.32, 0.22]
Experiment 2	0.01 [-0.64, 0.68]	0.04 [-0.53, 0.60]

Note: 95% confidence intervals for Tobit estimator computed using bootstrap with 2,000 replicates. OLS intervals are normal-approximation based.

[3] Applying the typical normalization gives the more concise presentation, $Y_i^{Obs} = \mathbb{1}\left[Y_i^{Obs} > 0\right] \cdot Y_i^*$

Bibliography

Amemiya, Takeshi. 1985. *Advanced econometrics.* Harvard university press.

Berinsky, Adam J., Gregory A. Huber and Gabriel S. Lenz. 2012. "Evaluating Online Labor Markets for Experimental Research: Amazon.com's Mechanical Turk." *Political Analysis* 20:351–368.

Bullock, John G. 2009. "Partisan bias and the Bayesian ideal in the study of public opinion." *The Journal of Politics* 71(03):1109–1124.

Coppock, Alexander. 2018. *ri2: Randomization Inference for Randomized Experiments.* R package version 0.1.1.
URL: *https://CRAN.R-project.org/package=ri2*

Davidson, Russell and James G MacKinnon. 2004. *Econometric theory and methods.* Vol. 5 Oxford University Press New York.

Dunn, Thomas J., Thom Baguley and Vivienne Brunsden. 2013. "From alpha to omega: A practical solution to the pervasive problem of internal consistency estimation." *British Journal of Psychology* .
URL: *http://dx.doi.org/10.1111/bjop.12046*

Gerber, Alan and Donald P. Green. 1998. "Rational Learning and Partisan Attitudes." *American Journal of Political Science* 42(2):794–818.

Gerber, Alan S and Donald P Green. 2012. *Field experiments: Design, analysis, and interpretation.* WW Norton.

Kelley, Ken and Keke Lai. 2012. *MBESS: MBESS.* R package version 3.3.3.
URL: *http://CRAN.R-project.org/package=MBESS*

Rose, Jonathan. 2014. *The public understanding of political integrity: The case for probity perceptions.* Springer.

3 Appendix A: Qualtrics Survey Instruments

All post-treatment questions about policy attitudes came after post-treatment questions about trust. The order was randomized within each group of post-treatment questions.

Pretreatment questions:

sex
What is your sex?
- ❍ Male (1)
- ❍ Female (2)

birthyr
What is your year of birth?

mstatus
Please indicate your marital status:
- ❍ Married (1)
- ❍ Separated (2)
- ❍ Divorced (3)
- ❍ Widowed (4)
- ❍ Never Married (5)

educ
What is the highest level of education you have completed?
- ❍ Less than High School (1)
- ❍ High School / GED (2)
- ❍ Some college but no degree (3)
- ❍ Associate degree in college - Occupational/vocational program (4)
- ❍ Associate degree in college - Academic program (5)
- ❍ Bachelor's degree (e.g. BA, AB, BS) (6)
- ❍ Masters degree (e.g. MA, MS, MEng, MEd, MSW, MBA) (7)
- ❍ Professional School Degree (e.g. MD, DDS, DVM, LLB, JD) (8)
- ❍ Doctorate degree (e.g. PhD, EdD) (9)

race
What is your race?
- ❍ White (1)
- ❍ Black or African American (2)
- ❍ American Indian or Alaska Native (3)
- ❍ Asian (4)
- ❍ Native Hawaiian or Other Pacific Islander (5)
- ❍ Hispanic (6)
- ❍ Other (7)

emp

What is your current employment status?

- ❍ Full time (working 35 or more hours per week) (1)
- ❍ Part time (working 1 to 34 hours per week) (2)
- ❍ Not employed (3)

partyid (partyid2 if ‘other’)

Generally speaking, do you usually think of yourself as a Republican, a Democrat, an independent, or what?

- ❍ Republican (1)
- ❍ Democrat (2)
- ❍ Independent (3)
- ❍ Something else (please specify) (4) ____________________

ideology

Generally speaking, would you describe your political views as very conservative, conservative, moderate, liberal or very liberal?

- ❍ Very conservative (1)
- ❍ Conservative (2)
- ❍ Moderate (3)
- ❍ Liberal (4)
- ❍ Very liberal (5)

housing (housing2 if ‘other’)

Does your family own or rent a home?

- ❍ Own home (1)
- ❍ Rent home (2)
- ❍ Live with parents/family (3)
- ❍ Other (4) ____________________

totincome
Which category represents your total combined income during the past 12 months? This includes money from jobs, net income from business, farm or rent, pensions, dividends, interest, social security payments and any other money income received.

- ❍ Less than $5,000 (1)
- ❍ 5,000 to 7,499 (2)
- ❍ 7,500 to 9,999 (3)
- ❍ 10,000 to 12,499 (4)
- ❍ 12,500 to 14,999 (17)
- ❍ 15,000 to 19,999 (7)
- ❍ 20,000 to 24,999 (8)
- ❍ 25,000 to 29,999 (9)
- ❍ 30,000 to 34,999 (10)
- ❍ 35,000 to 39,999 (11)
- ❍ 40,000 to 49,999 (12)
- ❍ 50,000 to 59,999 (13)
- ❍ 60,000 to 74,999 (14)
- ❍ 75,000 to 99,999 (15)
- ❍ 100,000 to 149,999 (16)
- ❍ 150,000 or more (6)

religion (religion2 if ‘other’)
Do you consider yourself Protestant, Roman Catholic, Jewish, or something else?

- ❍ Protestant (1)
- ❍ Catholic (2)
- ❍ Jewish (3)
- ❍ Atheist (4)
- ❍ Other (5) ____________________

region
In which region of the United States do you live?

- ❍ Northeast (1)
- ❍ Midwest (2)
- ❍ South (3)
- ❍ West (4)

lendmoney
How often do you lend money to friends?
- ❍ Very often (1)
- ❍ Often (2)
- ❍ Sometimes (3)
- ❍ Rarely (4)
- ❍ Never (5)

lendposs
How often do you lend personal possessions to friends?
- ❍ More than once a week (1)
- ❍ About once a week (2)
- ❍ About once a month (3)
- ❍ Once a year or less (4)
- ❍ Never (5)

doorlock
How often do you intentionally leave your door unlocked (when nobody is home)?
- ❍ Very often (1)
- ❍ Often (2)
- ❍ Sometimes (3)
- ❍ Rarely (4)
- ❍ Never (5)

Posttreatment Questions:

persuade
How persuasive would you characterize the article you just read?
- ❍ Very persuasive (4)
- ❍ Somewhat persuasive (5)
- ❍ Neither persuasive or unpersuasive (6)
- ❍ Somewhat unpersuasive (7)
- ❍ Very unpersuasive (8)

changeview
Would it be fair to say the article you just read changed your views about the topic discussed?
- ❍ Yes (1)
- ❍ No (2)

Trust Questions (Order Randomized):

gentrust1
Some people say that most people can be trusted. Others say you can’t be too careful in your dealings with people. How do you feel about it?
- ❍ You can't be too careful (2)
- ❍ Most people can be trusted (1)

gentrust2
Would you say that most people are more inclined to help others or more inclined to look out for themselves?
- ❍ More inclined to look out for themselves (2)
- ❍ More inclined to help others (1)

gentrust3
If you don’t watch yourself, people will take advantage of you
- ❍ Agree (1)
- ❍ Disagree (4)

gentrust4
No one is going to care much what happens to you, when you get right down to it.
- ❍ Agree (1)
- ❍ Disagree (2)

gentrust5
Human nature is fundamentally cooperative.
- ❍ Agree (1)
- ❍ Disagree (2)

Please indicate how much you agree or disagree with the following statements:

	Strongly Disagree (1)	Moderately Disagree (2)	Slightly Disagree (3)	Slightly Agree (4)	Moderately Agree (5)	Strongly Agree (6)
We generally cannot trust politicians. (**govtrust1**)	❍	❍	❍	❍	❍	❍
People in government are too often interested in looking after themselves. (**govtrust2**)	❍	❍	❍	❍	❍	❍
Government is run by a few big interests who look after their own interests. (**govtrust3**)	❍	❍	❍	❍	❍	❍
A lot of politicians are corrupt (**govtrust4**)	❍	❍	❍	❍	❍	❍

trustfed
How much of the time do you think you can trust the government in Washington to do what is right?
- ❍ Always (1)
- ❍ Most of the time (2)
- ❍ About half the time (3)
- ❍ Some of the time (4)
- ❍ Never (5)

truststate
How much of the time do you think you can trust the government in your state to do what is right?
- ❍ Always (1)
- ❍ Most of the time (2)
- ❍ About half the time (3)
- ❍ Some of the time (4)
- ❍ Never (5)

In the next section you will be asked whether you trust a series of groups and institutions. For each group presented please indicate whether you: 1) Trust the group in question. That is, you expect the group will generally do the right thing. or 2) Distrust the group in question. That is, you expect the group will generally not do the right thing.

Please indicate whether you trust or distrust the following group:

	Trust (1)	Distrust (2)
Family members (**trustfam**)	❍	❍

Please indicate whether you trust or distrust the following group:

	Trust (1)	Distrust (2)
Friends (**trustfriend**)	❍	❍

Please indicate whether you trust or distrust the following group:

	Trust (1)	Distrust (2)
Doctors (**trustdr**)	❍	❍

Please indicate whether you trust or distrust the following group:

	Trust (1)	Distrust (2)
Scientists (**trustscience**)	❍	❍

Please indicate whether you trust or distrust the following group:

	Trust (1)	Distrust (2)
People in your neighborhood (**trustneighbor**)	❍	❍

Please indicate whether you trust or distrust the following group:

	Trust (1)	Distrust (2)
Universities (**trustuni**)	❍	❍

Please indicate whether you trust or distrust the following group:

	Trust (1)	Distrust (2)
The federal government in Washington (**trustfed2**)	❍	❍

Please indicate whether you trust or distrust the following group:

	Trust (1)	Distrust (2)
Your state government (**truststate2**)	❍	❍

Please indicate whether you trust or distrust the following group:

	Trust (1)	Distrust (2)
Your local government (**trustlocal**)	❍	❍

Please indicate whether you trust or distrust the following group:

	Trust (1)	Distrust (2)
The American media (**trustmedia**)	❍	❍

Please indicate whether you trust or distrust the following group:

	Trust (1)	Distrust (2)
Strangers (**truststranger**)	❍	❍

Please indicate whether you trust or distrust the following group:

	Trust (1)	Distrust (2)
The police in your area (**trustpolice**)	❍	❍

Please indicate whether you trust or distrust the following group:

	Trust (1)	Distrust (2)
Government administrators (**trustadmin**)	❍	❍

Please indicate whether you trust or distrust the following group:

	Trust (1)	Distrust (2)
Politicians (**trustpols**)	❍	❍

Now we would like to ask you some questions about public office holders as a whole – by this we mean heads of state and national government, Congress men and women, and public officials with jobs in government departments, local councils or other public bodies

probity1
Overall, how would you rate the standards of conduct of public office holders in the USA?
❍ Very low (1)
❍ Quite low (2)
❍ Neither high nor low (3)
❍ Quite high (4)
❍ Very high (5)

probity2
And how do you think standards of public office holders in the USA today compare with a few years ago?
❍ Got a lot worse (1)
❍ Got a bit worse (2)
❍ Stayed the same (3)
❍ Improved a little (4)
❍ Improved a lot (5)

probity3

How confident do you feel that the authorities in the USA are committed to improving standards in public life?
- ❍ Not at all confident (1)
- ❍ Not very confident (2)
- ❍ Don't know (4)
- ❍ Fairly confident (5)
- ❍ Very confident (6)

probity4
And how confident do you feel that the authorities will generally uncover wrongdoing by people in public life?
- ❍ Not at all confident (1)
- ❍ Not very confident (2)
- ❍ Don't know (3)
- ❍ Fairly confident (4)
- ❍ Very confident (5)

Probity5
And when people in public life are caught doing wrong, how confident do you feel that the authorities will punish them?
- ❍ Not at all confident (1)
- ❍ Not very confident (2)
- ❍ Don't know (3)
- ❍ Fairly confident (4)
- ❍ Very confident (5)

Please say how many American politicians you think actually do these things.

	None (1)	A few (2)	About half (3)	Most (4)	All (5)
They are dedicated to doing a good job for the public (**perf1**)	❍	❍	❍	❍	❍
They use their power for their own personal gain (**perf2**)	❍	❍	❍	❍	❍
They take bribes (**perf3**)	❍	❍	❍	❍	❍
They own up when they make mistakes (**perf4**)	❍	❍	❍	❍	❍
They explain the reasons for their actions and decisions (**perf5**)	❍	❍	❍	❍	❍
They make sure public money is used wisely (**perf6**)	❍	❍	❍	❍	❍
They are in touch with what the general public thinks is important (**perf7**)	❍	❍	❍	❍	❍
They are competent at their jobs (**perf8**)	❍	❍	❍	❍	❍

Policy Questions (Order Randomized):

genspend
Some people think the government should provide fewer services, even in areas such as health and education, in order to reduce spending. Suppose these people are at one end of a scale, at point 1. Other people feel that it is important for the government to provide many more services even if it means an increase in spending. Suppose these people are at the other end, at point 7. And, of course, some other people have opinions somewhere in between, at points 2,3,4,5 or 6. Where would you place yourself on this scale, or haven't you thought much about this?

- ❍ Cut Services/Spending 1 (1)
- ❍ 2 (2)
- ❍ 3 (3)
- ❍ 4 (4)
- ❍ 5 (5)
- ❍ 6 (6)
- ❍ More services/spending 7 (7)
- ❍ Don't know; haven't thought about it much (8)

livingstd
Some people feel that the government in Washington should see to it that every person has a job and a good standard of living. Suppose these people are at one end of a scale, at point 1. Others think the government should just let each person get ahead on his/her own. Suppose these people are at the other end, at point 7. And, of course, some other people have opinions somewhere in between, at points 2,3,4,5 or 6. Where would you place yourself on this scale, or haven't you thought much about this?

- ❍ Every person has a job and a good standard of living 1 (1)
- ❍ 2 (2)
- ❍ 3 (3)
- ❍ 4 (4)
- ❍ 5 (5)
- ❍ 6 (6)
- ❍ Let each person get ahead on their own 7 (7)
- ❍ Don't know; haven't thought about it much (8)

minorityaid

Some people feel that the government in Washington should make every effort to improve the social and economic position of blacks. Suppose these people are at one end of a scale, at point 1. Others feel that the government should not make any special effort to help blacks because they should help themselves. Suppose these people are at the other end, at point 7. And, of course, some other people have opinions somewhere in between, at points 2,3,4,5 or 6. Where would you place yourself on this scale, or haven't you thought much about it?

- ❍ Government should help blacks 1 (1)
- ❍ 2 (2)
- ❍ 3 (3)
- ❍ 4 (4)
- ❍ 5 (5)
- ❍ 6 (6)
- ❍ Blacks should help themselves 7 (7)
- ❍ Don't know; haven't thought about it much (8)

insurance

There is much concern about the rapid rise in medical and hospital costs. Some people feel there should be a government insurance plan which would cover all medical and hospital expenses for everyone. Suppose these people are at one end of a scale, at point 1. Others feel that medical expenses should be paid by individuals, and through private insurance plans like Blue Cross or some other company paid plans. Suppose these people are at the other end, at point 7. And of course, some people have opinions somewhere in between at points 2,3,4,5 or 6. Where would you place yourself on this scale, or haven't you thought much about this?

- ❍ Government insurance plan 1 (1)
- ❍ 2 (2)
- ❍ 3 (3)
- ❍ 4 (4)
- ❍ 5 (5)
- ❍ 6 (6)
- ❍ Private insurance plan 7 (7)
- ❍ Don't know; haven't thought about it much (8)

women

Recently there has been a lot of talk about women’s rights. Some people feel that women should have an equal role with men in running business, industry and government. Suppose these people are at one end of a scale, at point 1. Others feel that a women's place is in the home. Suppose these people are at the other end; at point 7. And of course, some people have opinions somewhere in between, at points 2,3,4,5, or 6. Where would you place yourself on this scale or haven’t you thought much about this?

- ❍ Women and men should have an equal role 1 (1)
- ❍ 2 (2)
- ❍ 3 (3)
- ❍ 4 (4)
- ❍ 5 (5)
- ❍ 6 (6)
- ❍ Women's place is in the home 7 (7)
- ❍ Don't know; haven't thought about it much (9)

defense

Some people believe that we should spend much less money for defense. Suppose these people are at one end of a scale, at point 1. Others feel that defense spending should be greatly increased. Suppose these people are at the other end, at point 7. And, of 4, , some other people have opinions somewhere in between, at points 2,3,4,5, or 6. Where would you place yourself on this scale or haven't you thought much about this?

- ❍ Greatly decrease defense spending 1 (1)
- ❍ 2 (2)
- ❍ 3 (3)
- ❍ 4 (4)
- ❍ 5 (5)
- ❍ 6 (6)
- ❍ Greatly increase defense spending 7 (7)
- ❍ Don't know; haven't thought about it much (9)

For each of the budget items below please indicate whether federal spending should be increased, decreased or kept about the same:

	Decreased (1)	Kept the same (2)	Increased (3)
Welfare programs (**redist1**)	❍	❍	❍
Foreign aid (**redist2**)	❍	❍	❍
Food stamps (**redist3**)	❍	❍	❍
Programs that assist blacks and other minorities (**redist4**)	❍	❍	❍
Programs that assist the homeless (**redist5**)	❍	❍	❍

For each of the budget items below please indicate whether federal spending should be increased, decreased or kept about the same:

	Decreased (1)	Kept the same (2)	Increased (3)
Highway construction (**dist1**)	❍	❍	❍
Social security (**dist2**)	❍	❍	❍
Programs that improve and protect the environment (**dist3**)	❍	❍	❍
Public schools (**dist4**)	❍	❍	❍
Crime prevention (**dist5**)	❍	❍	❍
Preventing illegal immigration (**dist6**)	❍	❍	❍
